# Implications of Exposure to Air Pollution on Male Reproduction: The Role of Oxidative Stress

**DOI:** 10.3390/antiox13010064

**Published:** 2024-01-01

**Authors:** Temidayo S. Omolaoye, Bongekile T. Skosana, Lisa Marie Ferguson, Yashthi Ramsunder, Bashir M. Ayad, Stefan S. Du Plessis

**Affiliations:** 1 College of Medicine, Mohammed Bin Rashid University of Medicine and Health Sciences, Dubai P.O. Box 505055, United Arab Emirates; stefan.duplessis@mbru.ac.ae; 2 Division of Medical Physiology, Faculty of Medicine and Health Sciences, Stellenbosch University, Tygerberg, Cape Town 7602, South Africa; bts@sun.ac.za (B.T.S.); 18546854@sun.ac.za (L.M.F.); 21911886@sun.ac.za (Y.R.); 3 Department of Physiology, Faculty of Medicine, Misurata University, Misratah P.O. Box 2478, Libya; b.ayad@med.misuratau.edu.ly

**Keywords:** air pollution, male infertility, semen abnormalities, oxidative stress, male sexual health

## Abstract

Air pollution, either from indoor (household) or outdoor (ambient) sources, occurs when there is presence of respirable particles in the form of chemical, physical, or biological agents that modify the natural features of the atmosphere or environment. Today, almost 2.4 billion people are exposed to hazardous levels of indoor pollution, while 99% of the global population breathes air pollutants that exceed the World Health Organization guideline limits. It is not surprising that air pollution is the world’s leading environmental cause of diseases and contributes greatly to the global burden of diseases. Upon entry, air pollutants can cause an increase in reactive oxygen species (ROS) production by undergoing oxidation to generate quinones, which further act as oxidizing agents to yield more ROS. Excessive production of ROS can cause oxidative stress, induce lipid peroxidation, enhance the binding of polycyclic aromatic hydrocarbons (PAHs) to their receptors, or bind to PAH to cause DNA strand breaks. The continuous and prolonged exposure to air pollutants is associated with the development or exacerbation of pathologies such as acute or chronic respiratory and cardiovascular diseases, neurodegenerative and skin diseases, and even reduced fertility potential. Males and females contribute to infertility equally, and exposure to air pollutants can negatively affect reproduction. In this review, emphasis will be placed on the implications of exposure to air pollutants on male fertility potential, bringing to light its effects on semen parameters (basic and advanced) and male sexual health. This study will also touch on the clinical implications of air pollution on male reproduction while highlighting the role of oxidative stress.

## 1. Introduction

Over the years, indicators of male fertility potential such as normal semen parameters are shown to be declining. Carlsen et al. reported a decrease in the average sperm count from 113 × 10^6^/mL in 1940 to 66 × 10^6^/mL in 1990 [1], and Swan et al. showed a 1.5% decrease per year in sperm count in the USA and 3% decline annually in Europe/Australia between 1934 and 1996 [2]. Levine et al., after analyzing semen samples collected from 42,935 fertile men over four decades retrospectively, reported a significant decline in global sperm concentration between 1973 and 2011 (0.70 million/mL/year) [3].

Since semen quality (motility, morphology, sperm concentration, sperm count) is declining and the socioeconomic burden of male infertility is increasing [4,5], it is paramount to investigate the causes. Some of the possible explanations for a decline in sperm quality and quantity include the development of cryptorchidism, hypospadias, and testicular cancer, thereby suggesting a shared prenatal etiology [5]. Lifestyle factors such as diet [6], stress [7], smoking [8], excessive exercise, and BMI [3,9,10] are also attributable causes of male infertility. In recent years, studies have documented the significant implications that environmental factors impose on male fertility potential. Such are the exposure to endocrine-disrupting chemicals [11,12], pesticides [13], heat [14], and air pollutants of varying size, intensity, and quantity [15].

Air pollution occurs when there is presence of respirable particles in the form of chemical, physical, or biological agents that modify the natural features of the atmosphere or environment. Air pollution is one the world’s leading environmental cause of diseases, as almost 2.4 billion people are exposed to hazardous levels of indoor pollution, while 99% of the global population breathes air pollutants that exceed the World Health Organization (WHO) guideline limits [16]. As a result, air pollution is responsible for over 6.4 million deaths every year [17]. In addition to this, the global burden of the diseases caused by air pollution is estimated at about USD 8.1 trillion a year, which is equivalent to 6.1% of the global gross domestic product (GDP) [18].

**Figure 1:**
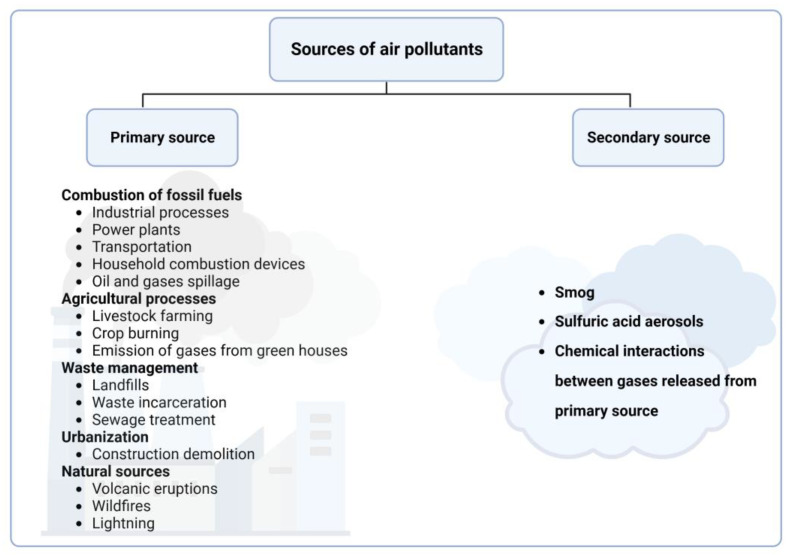
Sources of air pollution. Briefly, air pollution can be of a primary or secondary source. The primary source emits pollutants directly into the environment, including the following: (i) combustion of fossil fuels, whereby pollutants are released from industrial processes like burning coal, energy production, electricity generation, and exhaust emissions from transportation devices; (ii) agricultural activities, whereby pollutants are emitted from animal waste, methane emission from livestock, and burning of crop residue and agricultural waste; (iii) waste management (emission from decomposing organic waste in landfills and burning of solid waste in incinerators); (iv) emission from manufacturing processes, e.g., metal and cement production; (v) dust and particulate matter released from demolition and construction activities; (vi) natural sources, including from the emission of gases, particulate matter, and aerosols during volcanic eruptions and wildfires. On the other hand, pollutants of secondary sources are produced through chemical reactions between primary pollutants. These include ground level ozone (O_3_), which is formed by the reaction between volatile organic compounds and nitrogen oxides in the presence of sunlight. Sulfuric acid aerosols are formed from sulfur dioxides reacting with water, while nitric acid is formed from nitrogen dioxides reacting with water. Smog is formed by the interaction between nitrogen oxides and volatile organic compounds in the presence of sunlight.

**Figure 2:**
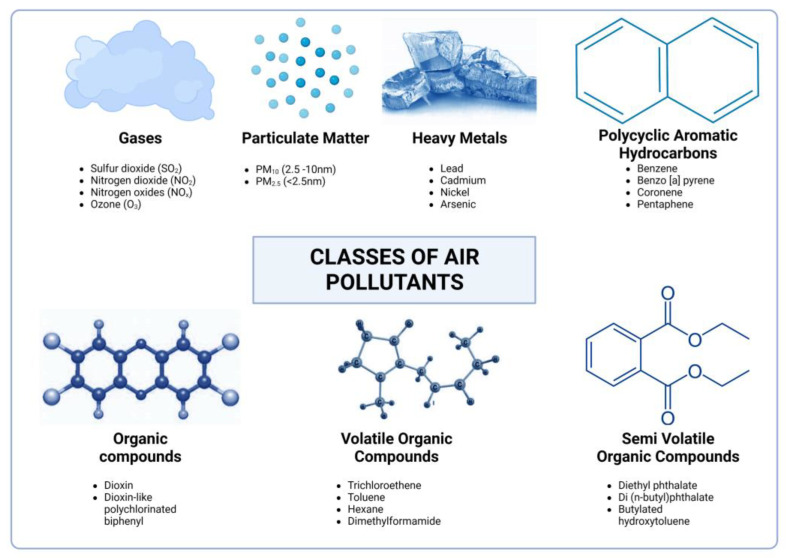
Classes and examples of air pollutants.

Exposure to air pollutants occurs continuously, either from indoor (household) or outdoor (ambient) sources, resulting in exposure to transient mixtures of pollutants. It is also known that some air pollutants transition between phases and are rather categorized as being part of a class of air pollutant that is readily measured. Thus, when it comes to air pollutant classification, many believe that there is no standardized way, making the classification process challenging and complicated. Nevertheless, in the process of developing standardized methods, a consensus has been reached regarding the grouping of air pollutants based on certain parameters: the phase of the air pollutant (gaseous, liquid, solid), the source (primary, secondary), the location (indoor, outdoor), the place (urban, rural), and the recognition that a pollutant can have multiple sources of emission. Therefore, air pollutants come from various sources and can be broadly categorized into primary (pollutants emitted directly from source) and secondary (derived from chemical reactions between gases) sources based on their origin and formation (Figure 1). Outdoor and indoor air pollutants can come from similar processes such as the incomplete combustion of fuels or chemical reactions between gases. Household activities such as boiling water for bathing, cooking animal fodder, or cooking and heating in general with dirty technologies, such as lighting with kerosene, emit a range of harmful pollutants indoors, while activities such as high-temperature combustion in vehicles, industries, and power-generating facilities contribute to ambient air pollution [16]. In lieu of this, air pollutants can likewise be of natural or anthropogenic origin [19,20]. Additionally, depending on the origin, phase, and source, air pollutants are divided into different types, including particulate matter (PM), ground-level ozone (O_3_), nitrogen oxides (NOx), sulfur dioxide (SO_2_), carbon monoxide (CO), volatile organic compounds (VOCs), heavy metals, ammonia (NH_3_), halogens, greenhouse gases, radon, aerosols and particulate matter precursors, polycyclic aromatic hydrocarbons, semi-volatile organic compounds, and organic compounds. These classifications and examples are briefly summarized in Figure 2.

As shown in Figure 2, several air pollutants belonging to diverse classes have been recognized to be hazardous. Nevertheless, based on available evidence and as reported by the WHO, air pollutants identified to cause public health concerns include the following: CO, nitrogen dioxide (NO_2_), SO_2_, O_3_, and PM (2.5 μm; 10 μm). These specific air pollutants will be the pivotal point for discussion in the current study. Health problems can occur due to short- and long-term exposure to these various pollutants.

It is well documented that exposure to PM, which refers to inhalable particles composed of sulphate, nitrates, ammonia, sodium chloride, black carbon, mineral dust, or water, having varied aerodynamic diameters (≤2.5 μm to 10 μm), is associated with morbidity and mortality from cardiovascular and respiratory diseases and cancer. Other pollutants have also been associated with diverse health problems [21,22]. Briefly, NO_2_, a water-soluble reddish brown gas, is a known oxidant having ambient and household sources. It is also an important precursor of O_3_, and it plays a role in the pathogenesis of diseases, especially respiratory disorders. O_3_ is another gas that is formed from photochemical reaction with other air pollutants such as volatile organic compounds, CO, and NOx emitted from vehicle exhaust and industry. Prolonged and excessive exposure to O_3_ is also implicated in respiratory disease progression. CO is known to diffuse across the lung tissues into the bloodstream, subsequently causing a drastic reduction in the availability of oxygen in the cell and resultantly driving disease pathogenesis. SO_2_, which is mainly derived from the combustion of fossil fuels, is also associated with respiratory disorders and other health problems. Details on the roles and implications of NO_2_, O_3_, SO_2_, and CO on general health and well-being were well described by the following authors [23,24,25,26,27,28].

Upon entry of air pollutants, either through dermal uptake, inhalation, or ingestion, their absorption and distribution depend on the mode of entry and solubility. Several studies have reviewed in detail the emission and re-emission implications, formation, mode of uptake, distribution, and transformation pathways of these air pollutants [29,30,31,32,33,34,35,36].

In accordance with the WHO diagnostic criteria for male infertility, assessing semen quality remains a gold standard. Similarly, medical history, physical examination, hormonal analysis, and other advanced parameters (such as sperm DNA fragmentation, immunological tests, assessment of oxidative stress biomarkers, acrosome reaction, and genetic and genomic tests) are also taken into consideration [37]. Emerging studies have examined whether exposure to air pollutants such as CO, NO_2_, SO_2_, O_3_, and PM would impair semen quality, and findings have been conflicting. Some studies reported the negative effects of air pollution on semen quality [38,39,40], while others have shown no association [41]. Najafi et al. in their meta-analysis revealed that air pollution can lead to reduced sperm motility, concentration, morphology, and semen volume [15]. Other studies have also reported similar findings [42]. Additionally, the occurrence of semen abnormalities related to exposure to PM of <10 μm and 2.5 μm (PM_10_ and PM_2.5_) is well documented; nevertheless, a study by Zhang et al. showed that men exposed to PM_1_ (particles with an aerodynamic diameter of 1μm) developed severe semen abnormalities [43]. Not only is air pollution able to cause semen abnormalities, studies have also shown its ability to cause sperm DNA fragmentation [44,45], chromatin damage [46,47], shorter telomere length [48,49], a lower Y:X sperm chromosome ratio [38], sperm aneuploidy, and other genetic abnormalities [45]. Among the mechanisms through which air pollutants negatively impact male fertility is the direct toxicity on the testicular microenvironment, which may act through the alteration of plasma membrane fluidity and electrochemical potential [50], alteration of mitochondrial function and upregulating pro-apoptotic genes [51], activation of the apoptotic pathway, and the initiation of oxidative stress. Therefore, this review aimed to systematically analyze the literature to gather evidence relating to the effects of air pollution (with emphasis on pollutants of public health concern) on male fertility at both the conventional and advanced levels and to elucidate their impact on male sexual health. This study will also touch on the clinical implications for air pollution on male reproductive health while highlighting the role of oxidative stress.

## 2. Search Methods

A systematic literature search was conducted on the different air pollutants of public health concern (CO, NO_2_, SO_2_, O_3_, and PM (2.5 μm; 10 μm)) as reported by the WHO. The literature search did not discriminate against papers published before a certain date. Evidence was retrieved from the diverse credible literature sources and electronic databases including PubMed, PubMed Central, Google Scholar, and UpToDate. Search terms such as “air pollutants” AND “male fertility” AND/OR “male reproduction” were used. The individual air pollutant of interest was matched with “spermatogenesis”, “semen”, “male sexual health”, and “reproduction”, etc.

The subsequent sections of this review present the findings from both animal and human studies demonstrating the effects of exposure to CO, NO_2_, SO_2_, O_3_, and PM (2.5 μm; 10 μm) on sperm formation, production, and semen quality (basic and advanced parameters). Furthermore, evidence highlighting the potential impact of these air pollutants on male sexual health and reproductive outcomes is also emphasized. Finally, the role of reactive oxygen species, the consequent development of oxidative stress, and how these influence the effects of the air pollutants on male reproduction are also discussed.

## 3. Implications of Exposure to Air Pollution on Male Reproductive Parameters

### 3.1. Evidence of the Effects of Air Pollution on Male Reproductive Parameters: Animal Studies

#### Basic and Advanced Parameters

Several animal studies have investigated the implications and consequences of exposure to air pollutants on male reproductive parameters. Reports from animal models assert that air pollutants decrease sperm concentration and motility; however, findings on the association between air pollutants and abnormal sperm morphology remain inconsistent [52,53,54,55,56,57,58]. A study by Cao et al. found that Sprague–Dawley rats exposed to PM_2.5_ daily, via intratracheal instillation for seven weeks, presented with a significantly reduced sperm count and increased abnormal morphology [54]. Another study observed the effect of PM_2.5_ exposure on sperm quality in a mouse model and reported that exposure to concentrated ambient PM_2.5_ decreased the sperm count in the epididymis [53]. However, no significant changes in sperm morphology were observed [53]. Moreover, Zhou et al. found that a time- and dose-dependent effect was observed in mice exposed to PM_2.5_ for 8 and 16 weeks, whereby, sperm count and motility were significantly reduced, and abnormal sperm morphology was significantly increased [57]. Conversely, Yang et al. found that despite the significant reduction in sperm count and motility after chronic exposure (29 weeks) to diesel exhaust PM_2.5_, no significant effects were observed on the sperm morphology of male mice [56]. It was therefore suggested that the inconsistent effects of exposure to PM_2.5_ observed on sperm morphology were dependent on the composition thereof and PM_2.5_ presumably impacted sperm morphology via alternative mechanisms to sperm count and motility [56]. Other studies have shown the adverse effects of exposure to PM_10_, NO_2_, SO_2_, CO, and NOx on total motility, total sperm count, sperm concentration, cell survival rate, DNA integrity, testicular histomorphology, and even on reproductive sex hormones [59,60,61,62]. In addition to the studies described above, several others have evaluated the mechanisms through which these pollutants of public health concern impact male reproduction. For instance, Meng and Bai reported an increase in testicular thiobarbituric acid reactive substance (TBARS) levels with subsequent decreases in superoxide dismutase (SOD) and glutathione peroxidase (GPx) activities following the exposure of mice to SO_2_ of varying concentrations for 30 days [63]. Similarly, Li et al. showed that as a repercussion of the imbalance between ROS production and antioxidant availability upon exposure to SO_2_, there was a decrease in the number of spermatogonia stem cells and subsequent reduction in sperm count [64]. A detailed explanation of the mechanisms through which these adverse effects are exerted is described later in this review. Summarized in Table 1 are findings from animal studies reporting the consequences of exposure to these air pollutants on male reproductive parameters.

In summary, many experimental studies have reported the detrimental effects of exposure to different concentrations of the mentioned air pollutants. Seasonality and duration of exposure are key factors influencing these effects. Adverse outcomes include diminished sperm quality, apoptosis of testicular germ cells, lowered sex hormone levels, decreased cell survival rate, and more. The implicated mechanism includes alteration of hormonal balance, excessive production of ROS and initiation of inflammation, reduced antioxidant bioavailability, and occurrence of oxidative stress.

### 3.2. Evidence of the Effects of Air Pollution on Male Reproductive Parameters: Human Studies

#### 3.2.1. Basic Parameters

Exposure to air pollution has been shown to negatively impact semen quality by reducing some basic parameters. These include semen volume, sperm concentration, total and progressive motility, and morphology [68,69,70,71], thus directly affecting reproductive potential in males. To understand the know-how through which air pollutants affect these parameters, numerous epidemiological and experimental studies have investigated the roles of seasonality (winter, summer, spring, autumn), air pollutant concentration, and duration of exposure. A study showed that there was no association between exposure to air pollutants and semen parameters during winter and summer months, while sperm motility and kinematic parameters were significantly reduced during spring and autumn months [46]. This shows that seasonality does play a role in the concentration of air pollutants and how the increase in air pollutants subsequently affected semen parameters adversely. Another study showed that although participants were exposed to the same concentration of PM_2.5_, nevertheless, semen parameters were reduced after two and three months of exposure [72]. This further shows that duration of exposure to air pollutants has a negative effect on semen parameters.

A recent review by Seli et al. investigated the impact of air pollution and endocrine disruptors on natural and assisted reproduction in both males and females. These authors found that common pollutants such as CO, NOx, NO_2_, O_3_, SO_2_, and PM were negatively associated with sperm parameters [73]. However, they noted that they were unable to perform direct experimental studies to determine dose–effect relationships for specific air pollutants [73]. Another study by Kumar et al. [74] found that although air pollution was detrimental to male fertility, more data are needed to draw specific conclusions and make assertions on the impact of exposure to air pollutants on male reproduction. Summarized in Table 2 are the reports from studies that examined the effects of the air pollutants of public health concern on basic sperm parameters.

Unfortunately, not only does exposure to these pollutants cause an adverse effect on the reproductive potential of the immediate generation, but also on the male offspring of those affected. Findings from human and animal studies alluded to this effect [67,75]. For instance, findings from a population-based study showed that maternal exposure to SO_2_ caused an epigenetic change in the male offspring [75]. Similar findings were reported from animal studies following maternal exposure to PM_2.5_, SO_2_, PM_10_, and NO_2_ [67].

**Table 2:** The effects of air pollutants of public health concern on basic human sperm parameters.

Reference	Type of Study Design	Country	Sample Size	Pollutants	Study Outcome
[46]	Cohort Study/Cross-sectional study	Czech Republic	54	PM_1O,_ SO_2,_ NO_2,_ CO, O_3_, Benzene, Benzo[a]pyrene	○ No significant association between air pollutants and sperm parameters was observed during the winter and summer months ○ Sperm kinetic parameters such as STR, VAP, VSL and LIN, and DSL were significantly reduced during the spring and autumn months
[68]	Prospective cohort study	China	15,112	PM_2.5_, PM_1O_	○ Exposure to PM_2.5_ and PM_1O_ significantly decreased sperm concentration, total sperm count, total motility, progressive motility, total motile sperm count, and progressively motile sperm count, during the entire spermatogenesis period
[69]	Longitudinal study	China	15,112	PM_2.5_, CO, SO_2_, NO_2_, O_3_	○ Exposure to ambient SO_2_ was negatively associated with all semen quality parameters except for the progressive motility ○ There was also a linear and non-linear dose–response association between SO_2_ and sperm motility
[70]	Retrospective cohort study	China	1168	PM_2.5_, PM_1O_, CO, SO_2_, NO_2_, O_3_	○ PM_2.5_ and PM_1O_ were shown to significantly decrease sperm count ○ A negative association between all air pollutants and sperm parameters was observed ○ O_3_ significantly reduced motility in the late stage of sperm development
[71]	Cross-sectional study/Comparative study	China	1346	PM_1O_, SO_2_, NO_2_	○ Exposure to these pollutants adversely affected sperm morphology sperm motility and sperm kinematics (VCL and VSL)
[72]	Retrospective cohort study	United States of America	1699	PM_2.5_	○ Negative correlation was observed between PM_2.5_ and motility after 2 and 3 months of exposure to pollutants
[76]	Retrospective study and longitudinal study	China	686	PM_2.5_, PM_1O_, CO, SO_2_, NO_2_, O_3_	○ Exposure to PM_2.5,_ PM_1O_, and CO negatively affected sperm concentration and the concentration of progressively motile spermatozoa
[77]	Prospective cohort study	China	3940	PM_2.5_, PM_1O_, CO, SO_2_, NO_2_	○ There was a decrease in total motility, progressive motility, and sperm morphology following exposures to all pollutants tested ○ The SO_2_-induced effects were mostly on sperm morphology
[78]	Retrospective cohort study/Cross-sectional study	Poland	327	PM_2.5_, PM_1O_, CO, SO_2_, NOx	○ Exposure to pollutants significantly altered sperm morphology
[79]	Retrospective cohort study	Italy	406	PM_2.5_, PM_1O_	○ PM_2.5_ was positively associated with total sperm count ○ PM_10_ was positively associated with both semen volume and percentage of normal sperm morphology
[80]	Prospective	China	1759	SO_2_, NO_2_, CO, O_3_	○ After 90 days of exposure to the pollutants, reported observations included the following: ○ Decreased sperm concentration ○ Reduced sperm count ○ Reduced total motile sperm count ○ Reduce total and progressive motility ○ SO_2_ had the most adverse effect on semen quality
[81]	Prospective	China	72,917	PM_2.5_, PM_1O_, CO, SO_2_, NO_2_, O_3_	○ Following exposure to the pollutants for 30 days, there was as significant decrease in serum sex hormone levels (follicle-stimulating hormone, luteinizing hormone, estradiol, prolactin, especially testosterone)
[82]	Observational	Czech Republic	2585 parental pairs	SO_2_	○ Exposure to SO_2_ for 4 months before conception until 2 years after delivery led to the following: ○ Decreased fecundity ○ Increased sperm abnormalities
[75]	Population-based case–control study		4371 male infants	Maternal exposure to SO_2_, PM_10_, NO_2_	○ Maternal exposure to SO_2_ during the 3 months before and the first and second months after conception increased the risk of hypospadias in male offspring
[83]	Observational	Taiwan	282 male patients with primary infertility	SO_2_, NOx, O_3_	○ Exposure to NO_2_ and SO_2_ was negatively associated with the seminal parameters (sperm count and concentration) and decreased testicular volume
[84]	Prospective	Human Sperm Bank	1515	PM_2.5_, PM_1O_, CO, SO_2_, NO_2_, O_3_	○ Exposure to the different pollutants over 90 days resulted in decreased progressive and total motility ○ Adversely affected sperm concentration and total sperm number
[85]	Prospective	China	1069	PM_10_, NO_2_, SO_2_, O_3_	○ Exposure to these air pollutants at varying spermatogenesis stages adversely affected spermatogenesis ○ PM_10_ was associated with declined sperm concentration, reduced sperm count, reduced total and progressive motility, and reduced sperm concentration ○ SO_2_ induced a decrease in progressive and total motility ○ NO_2_ and O_3_ were not associated with the sperm quality parameters measured
[86]	Prospective	USA	5134	PM_10_, NO_2_, SO_2_, O_3_	○ Exposure to the different air pollutants resulted in a significant negative correlation between O_3_ levels (at 0–9, 10–14, and 70–90 days before donation) and sperm concentration, which was maintained after correction for the donor’s birth date, age at donation, temperature, and seasonality ○ Exposure to ambient O_3_ levels adversely affected semen quality, while no other pollutant was significantly associated with sperm quality outcomes
[87]	Cross-observational	China	1852	SO_2_	○ Exposure to SO_2_ and NO_2_ for 90 days significantly decreased sperm concentration, sperm count, and progressive motility ○ There was no association between exposure to SO_2_ and NO_2_ and semen volume

These studies postulated that the type of air pollutant (PM_2.5_, PM_10_, NO_2_, etc.), geographical area, time of the year (seasonality), concentration of the air pollutant, duration of exposure, and ambient temperature all affected the outcome on the semen parameters when two or more pollutants were combined, as opposed to directly influencing specific parameters individually. PM_2.5_ = particulate matter ≤ 2.5 μm in aerodynamic diameter; PM_10_ = particular matter < 10 μm in aerodynamic diameter; CO = carbon monoxide; NO_2_ = nitrogen dioxide; NO_x_ = nitric oxides; SO_2_ = sulfur dioxide; O_3_ = ozone; CASA = computer-aided sperm analysis; VCL = curvilinear velocity; VSL = straight line velocity; LIN = linearity; STR = straightness; DSL = length of straight line path.

#### 3.2.2. Advanced Parameters

Advanced semen parameters are described as tests performed in extension to routine semen analyses [37], which may be useful in the diagnosis of unexplained male infertility. These include the assessment of parameters such as sperm DNA fragmentation, genetic/genomic tests, ROS and oxidative stress, acrosome reaction, and sperm chromatin [37,88].

DNA damage is caused by several environmental carcinogens, resulting in mutations and other alterations to the genome [89]. Pollution has been shown to cause DNA–protein crosslinks, a common form of DNA damage, which occurs when reactive substances from the environment incite DNA-binding proteins to become trapped on DNA, making DNA repair extremely difficult. In addition, these DNA–protein crosslinks interfere with transcription and replication machinery [90].

The inability to repair DNA damage can lead to DNA fragmentation. Sperm DNA fragmentation has been hypothesized to be an early indicator of pollution-induced DNA damage and therefore regarded as a biomarker of sperm quality [39,91,92]. A study by Bergamo et al. assessed the effects of 22 potentially toxic chemical pollutants on sperm DNA, including aluminum (Al), arsenic (As), barium (Ba), and lead (Pb) quantified from the blood and seminal plasma of Italian men living in the metropolitan area of Naples. They reported the sperm DNA fragmentation index (DFI) to be significantly higher in participants with higher levels of environmental chemicals within their blood and seminal plasma [39]. Similarly, in the Czech Republic, air pollution as a result of coal combustion was comprehensively monitored by the country’s Teplice Program and significant associations were found between high levels of pollutants (PM_10_, SO_2_, NOx, CO) in the winter period and sperm DNA fragmentation, as well as changes in sperm chromatin structure [93]. This is supported by a study conducted amongst Polish men which showed that exposure to PM_2.5_ and PM_10_ increased their percentage of cells with immature chromatin, indicating DNA damage [78].

Environmental pollution has additionally been implicated in the higher percentages of sperm aneuploidy (abnormal chromosome number) in humans compared to other animals [94,95]. Exposure to reproductive toxicants from the environment induces structural changes within sperm chromosomes such as breaks and translocations, as well as abnormal chromosome numbers [96]. Aneuploidy has been noted as the most common chromosomal abnormality, and exposure to numerous air pollutants has been demonstrated to induce chromosomal abnormalities in germ cells [94]. The reason for this is the increased rate of non-disjunction events that occurs due to the presence of DNA–protein crosslinks, instigated largely by environmental pollutants [97].

In addition, sex chromosomes are more commonly damaged in relation to autosomal chromosomes [94,98]. A study constituting Polish men attending a fertility clinic for infertility assessment showed that exposure to PM_2.5_ was positively associated with chromosome disomy, particularly sex chromosome disomy, disomy Y, and disomy 21 [94,98]. Recio et al. demonstrated that organophosphorus pesticides were positively associated with sex null aneuploidies (the absence of sex chromosomes) in nine male members of an agricultural community in Mexico with no history of chronic illness, chemotherapy, or radiation [98]. It is essential to minimize sperm aneuploidies because an abnormal number of chromosomes within germ cells leads to pregnancy loss, growth retardation, birth defects, infertility, and prenatal mortality.

Furthermore, air pollution can alter the expression and function of genes in various tissues and organs. One of the mechanisms through which pollution can affect gene regulation is epigenetics, which refers to the heritable changes in gene activity that do not involve changes in the DNA sequence [99]. Epigenetic modifications such as DNA methylation and histone modifications can influence the accessibility and transcription of genetic material in response to environmental stimuli [100]. For instance, air pollution (PM_2.5_) can induce global and gene-specific changes in DNA methylation patterns in spermatozoa, which may affect the expression of genes involved in inflammation, oxidative stress, and DNA repair [101]. Although much is not known about whether the air pollutants of public health concern have a negative impact on genetic expression and regulation, what is known is that heavy metals, such as arsenic and cadmium, can also alter DNA methylation and histone acetylation levels in various tissues, which may disrupt the regulation of genes related to the cell cycle, apoptosis, and detoxification [102]. Additionally, endocrine disruptors, such as bisphenol A and phthalates, can interfere with the hormonal signaling pathways that modulate epigenetic enzymes and chromatin remodeling factors, which may lead to aberrant gene expression and epigenetic reprogramming in reproductive cells and tissues [103]. Environmental pollution can also induce genetic variations, such as mutations and copy number variations, which can alter the structure and function of genes, increasing susceptibility to diseases [104]. For instance, air pollution can increase the frequency of mutations in blood cells and sperm cells, which may contribute to the development of leukemia, lymphoma, and congenital malformations [105]. Heavy metals can also induce mutations and copy number variations in various tissues, which may affect the function of genes involved in DNA repair, cell cycle control, and tumor suppression [57,102].

Exposure to air pollutants can also affect the length and integrity of telomeres, which are the protective caps located at the ends of chromosomes and prevent DNA damage and chromosomal instability [106]. Telomere length is considered a biomarker of aging and disease risk, as it reflects the cumulative exposure to oxidative stress and inflammation [107]. Several studies have shown that exposure to air pollutants and endocrine disruptors can shorten telomere length in sperm cells, which may increase the risk of infertility [48]. Such is a study by Song et al. who showed that upon maternal exposures to PM_2.5_, PM_10_, SO_2_, CO, and NO_2_, there was a significant reduction in the telomere lengths of newborns, which was more evident in male infants [49]. Therefore, pollution-induced telomere shortening can be transmitted to the next generation through paternal inheritance, which may have adverse effects on offspring health and their lifespan [108].

## 4. Implications of Air Pollution on Male Sexual Health

Air pollution is an emerging environmental factor that is opined to predispose males to infertility [44,109]. Studies have reported an association between erectile dysfunction and environmental toxins [74,110,111]. Erectile dysfunction is the failure to attain and sustain a penile erection that is sufficient for the duration of sexual intercourse [112]. The etiology of erectile dysfunction is multifactorial; however, depression and anxiety are strong contributing factors [113]. More recently, air pollution was investigated as a contributing factor to developing erectile dysfunction. A study by Tallon et al. investigated whether exposure to PM_2.5_, SO_2_, NO_2_, CO, and O_3_ would affect the incidence of erectile dysfunction [114]. Participants were between the ages of 57 and 85 and were required to fill out a self-reported questionnaire on their erectile dysfunction status [114]. The authors found a positive correlation between exposures to PM_2.5_, NO_2_, and O_3_ and developing erectile dysfunction. However, the odd ratios of erectile dysfunction incident for each of the examined exposure times, including 1-to-7-year relocation averages, did not reach significance. Nevertheless, exposures to each pollutant were consistently associated with higher odds of developing erectile dysfunction [114]. Wang et al. conducted a similar study in an animal model. The study postulated that the observed increase in impotence post exposure to PM_2.5_ may be attributed to the decrease in both endothelial nitric oxide synthase (NOS) and NOS activity in the penile cavernous tissue [115]. Another rat study examined the effects of exposure to vehicle exhaust on erectile function in vivo and found a harmful effect of vehicle exhaust emissions on penile erection [111]. Although not yet regarded as a pollutant of public health concern, Bisphenol A is another commonly produced air pollutant [116]. An occupational cohort study also revealed that workers from BPA-exposed factories had a higher risk of developing male sexual dysfunction across all domains of male sexual function [110].

A study by Yang et al. investigated whether there is an association between long-term combined exposure to multiple air pollutants (PM_2.5_, PM_10_, SO_2_, and NO_2_) and the incidence of depression and anxiety. Their findings showed that indeed, long-term exposure to multiple air pollutants was associated with increased risk of depression and anxiety, with men showing a higher tendency to develop anxiety [117].

However, findings from animal models cannot be directly translated in that the mode of exposure may not necessarily represent the natural state of ambient or household air pollution [74]. Nevertheless, studies conducted amongst human participants showed that exposure to air pollutants can affect male sexual health, either via the initiation of depression and anxiety or via disruption of chemical pathways.

## 5. Effects of Air Pollution on Reproductive Outcomes

As previously discussed, it is imperative to note that air pollution has a significant deleterious impact on a wide range of relevant semen quality markers, which are crucial in determining the fertilizing ability of human spermatozoa. Several studies have sought to explore the possible relationships between various forms of gaseous substances and particulate matters present in ambient air and the potential of fertilization and reproductive outcomes. However, findings have shown a lack of consistency. For instance, a retrospective cohort study carried out in Shanghai, China showed that exposure to ambient air pollutants, mainly NO_2_ and PM_10_, was associated with lower odds of pregnancy and live births in IVF patients [118]. Another study conducted across multiple centers also in China evaluating the impact of air pollution and meteorological factors on IVF outcomes revealed that exposures to increased levels of PM_2.5_, SO_2_, and O_3_ were adversely associated with pregnancy outcomes in fresh IVF cycles. Moreover, the study found that these associations were influenced by air temperature, relative humidity, and wind speed [119]. Furthermore, in a large-scale retrospective cohort study conducted in the USA, acute and subacute exposures to PMs were shown to be associated with increased risk of miscarriage and reduced clinical pregnancy rates [120]. As reviewed by Carré et al., exposure to air pollution elicits multiple changes in cellular and physiological functions, which could potentially account for the deterioration observed in reproductive outcomes [121]. These changes include endocrine-disrupting activity and induction of oxidative stress, as well as DNA adducts and epigenetic modifications.

On the other hand, a lack of meaningful association between traffic-related air pollution exposure and fertility-assisted births was reported in a retrospective study conducted by Thampy and Vieira, based on data attained from the Massachusetts state birth registry [122]. However, they found that fertility-assisted births were associated negatively with traffic density and positively with distance from major roadways [122]. Furthermore, a similar study utilizing national data in the USA did not show any correlation between reproductive outcomes and average daily concentrations of PM_2.5_ and O_3_ [123]. Likewise, no clear evidence to support the link between IVF outcomes and exposure to O_3_ was found in another retrospective study conducted in Korea [124].

Taken together, the disparities in findings across the above cited studies can be explained by a number of factors, including the following: (i) These studies relied on data of air pollution measurements obtained from monitoring stations, which may not precisely reflect individual exposures. (ii) The retrospective nature of most of these studies suggests that the observed associations might have been confounded by other potential factors. (iii) It remains unclear whether these findings possess a quality of generalizability to other populations with varying racial/ethnic and socioeconomic backgrounds in diverse regions around the world, where susceptibility and patterns concerning air pollutants may differ [21]. While the resounding messages conveyed by these studies highlight the adverse impacts that air pollution might have on a diverse range of reproductive outcomes, it is imperative to emphasize the necessity for increasing awareness among the public and the authorities. Future studies are still warranted to better illuminate the correlations and potential mechanisms through which air pollution affects clinical fertilization and pregnancy outcomes.

## 6. Mechanisms through Which Air Pollutants Affect Sperm Parameters

Several theories and hypotheses have been postulated and experimented upon to comprehend the various adverse effects of air pollutants, not only on male reproduction but also on overall well-being. It is well established that air pollution has detrimental impacts on sperm parameters. However, the precise molecular mechanisms have not been thoroughly elucidated. This review will shed light on the investigated mechanisms.

**Figure 3:**
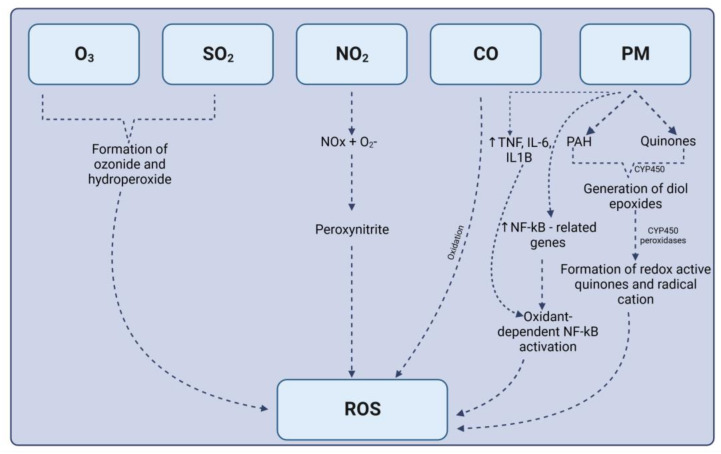
Formation of reactive oxygen species from the air pollutants of interest. Upon entry of O_3_ and SO_2_, they undergo numerous chemical reactions to form ozonide and hydroperoxide, which are ROS precursors. O_3_ induces intracellular oxidative stress through the formation of ozonide and hydroperoxide. This mechanism of oxidative damage involves the activation of Nrf2, heat shock protein 70, NF-κB, and an increase in the expression of various proinflammatory cytokines. Under environmental conditions, ozone rapidly transforms nitric oxide into NO_2_. Subsequent oxidation of NO_2_ leads to the production of peroxynitrite, a known precursor of reactive oxygen species (ROS). CO can penetrate alveoli, diffusing into the pulmonary circulation and interacting with free radicals, resulting in ROS production. Particulate matter (PM) can undergo various processes that convert it into ROS. Exposure to PM can lead to an increased expression of NF-kB-related genes, facilitating oxidant-dependent NF-kB activation. Activation of this pathway recruits proinflammatory cytokines, further amplifying ROS production.

Upon entry of air pollutants, either through dermal uptake, inhalation, or ingestion, their absorption and distribution depend on the mode of entry and solubility. For instance, for inhaled gases, high-affinity water-soluble gases such as SO_2_ are quickly removed by the upper respiratory airways, while those of moderate solubility such as O_3_ can reach the tracheobronchial tree where they can react with the mucous layer and subsequently damage the underlying tissue of the bronchioles. Conversely, gases with low aqueous solubility but reactivity with hemoglobin, such as CO, can penetrate further into the alveoli and diffuse into the pulmonary circulation to interact with free radicals to cause the production of ROS (Figure 3).

For entry through dermal uptake, upon accumulation of particles on the skin surface, oxidation occurs to generate quinones. Quinones are oxidized derivatives of reactive aromatic compounds, and they can act as oxidizing agents which can aid in the generation of ROS. Prolonged exposure leads to a further increase in the production of ROS, which subsequently may result in an imbalance in the production of ROS and the rate at which they are being scavenged or neutralized by the antioxidant system; hence, oxidative stress ensues. Entry through ingestion is usually via the production of protein crosslinks that occur during the processing of foods such as burning or boiling. Hence, exposure to air pollutants beyond the WHO guideline limits can alter sperm quality, with prolonged and continuous exposures having a greater impact, resulting in the exacerbation of existing pathologies.

The most explored mechanisms are inflammation, the induction of apoptosis, destruction or injury of the blood–testis barrier, and oxidative stress [125,126,127]. Oxidative stress is defined as the state of imbalance in the production of ROS and the activity of scavenging antioxidants [128]. It is well established that increased ROS generation can compromise fertilizing capacity by reducing sperm count and sperm motility [129,130,131,132]. Additionally, increased levels of antioxidants, particularly SOD and catalase (CAT), have been shown to inhibit several sperm functions by causing an imbalance between oxidation and reduction [132,133].

The occurrence of oxidative stress via various mechanisms of ROS production has been described as directly linked to the toxicity of air pollutants such as PM, with gaseous toxicants further exacerbating the effects [127,134,135]. ROS can interact with the polyunsaturated lipids on the plasma membrane to initiate lipid peroxidation which aids lipid and transmembrane protein breakdown, thus causing tissue injury [136]. ROS can also stimulate the release of proinflammatory cytokines, which results in the accumulation of neutrophils and other phagocytic cells that further generate ROS, thereby resulting in inflammation and metabolic injuries. The ROS-mediated mitogen-activated protein kinase (MAPK) signaling pathway was directly linked to PM_2.5_ exposure as observed by marked increases in the phosphorylation of these kinases, which in turn disrupted the blood–testis barrier integrity leading to impaired semen quality [134]. Furthermore, a study by Cao et al. [54] found that excessive ROS production within the testis was generated via the phosphatidylinositol 3-kinase (PI3K) and protein kinase B (Akt) signaling pathways, with elevated production promoted by phosphorylated Akt. The authors concluded that PM_2.5_ exposure leads to oxidative stress impairment via the PI3K/Akt signaling pathway, which ultimately disrupted spermatogenesis in male rats [54]. Additionally, excessive generation of ROS can cause DNA damage by modifying or breaking DNA strands [105]. These DNA lesions can result in base substitutions, insertions, deletions, or rearrangements which can affect the coding or regulatory regions of genes.

**Figure 4:**
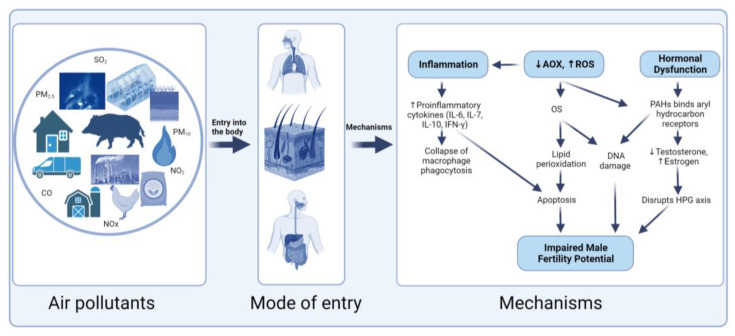
Mechanisms through which air pollutants affects male fertility. Upon entry of air pollutants through inhalation, percutaneous contact, or ingestion, there is an increase in reactive oxygen species (ROS) production. Excessive production of ROS can cause the recruitment of proinflammatory cytokines to cause inflammation and oxidative stress, induce lipid peroxidation, enhance the binding of polycyclic aromatic hydrocarbons (PAHs) to their receptors, or bind to PAH to cause DNA strand breaks and induce apoptosis. All these pathways aid in the pathogenesis of impaired male fertility potential. Air pollutants also act by disrupting hormone regulation, which can lead to impaired sperm quality. OS = oxidative stress; AOX = antioxidant; CO = carbon monoxide; NO_2_ = nitrogen dioxide; SO_2_ = sulfur dioxide; O_3_ = ozone; PM = particulate matter.

Additionally, studies have shown that exposure to air pollutants including PM_2.5_, PM_10_, SO_2_, NO_2_, and CO negatively affected the level of testosterone in the semen [78]. Hence, it can be deduced that prolonged exposure to air pollutants can alter the gonadal hormone regulation pathway, which may in turn affect fertility potential in males. Summarized in Figure 4 are the different mechanisms through which air pollutants can alter male fertility potential.

## 7. Conclusions

Air pollution, which is one of the world’s leading environmental causes of diseases, has been elucidated to adversely affect male fertility potential. These negative effects impact basic sperm parameters such as sperm concentration, sperm motility, morphology, and semen volume and advanced sperm parameters including DNA fragmentation, DNA damage, improper chromatin packaging, reduced telomere length, aneuploidy, genetic variation, and aberration. Upon entry through inhalation, percutaneous contact, or ingestion, they cause an increase in ROS production. Excessive production of ROS leads to the recruitment of proinflammatory cytokines to induce inflammation, oxidative stress, and lipid peroxidation, enhance the binding of polycyclic aromatic hydrocarbons (PAHs) to their receptors, or bind to PAH to cause DNA strand breaks and induce apoptosis. All these pathways aid in the pathogenesis of impaired male fertility potential. Additionally, studies have shown that exposure to air pollutants can affect male sexual health, either via the initiation of depression and anxiety or via the disruption of chemical pathways. Taken together, these studies highlight the adverse impacts that air pollution might impose on a diverse range of reproductive outcomes. Therefore, it is imperative to emphasize the necessity of increasing awareness among the public and the authorities. Future studies are recommended to investigate and validate mechanisms through which exposure to air pollutants adversely affects male fertility potential and at a larger scale, male reproduction.

## Figures and Tables

**Table 1:** The effects of air pollutants of public health concern on sperm parameters using animal models.

Reference	Type of Study Design	Model	Pollutants	Pollutant Concentrations and Duration	Sample Size	Study Outcome
[63]	Experimental	Mice	SO_2_	22, 56, and 112 mg/m^3^ administered at 6 h/day for 7 days	30	○ Exposure to varying concentrations of SO_2_ for 7 days, administered 6 h/day resulted in the following: ○ Increased levels of testicular thiobarbituric acid reactive substances (TBARSs) ○ Decreased testicular SOD and GPx activities
[65]	Experimental	Mice	SO_2_	3 h/day	48	○ Exposure to SO_2_ significantly decreased sperm quality and altered the BTB morphology and other ultrastructure of testis ○ Also observed was reduced mRNA expression of testicular occludin, claudin-11, ZO-1, N-cadherin, α-catenin, and connexin-43
[64]	Experimental	Mice	SO_2_, Arsenic	5 mg/m^3^ and/or 5 mg/L of arsenic		○ Exposure to both SO_2_ and arsenic: ○ Reduced sperm count ○ Increased percentage of sperm malformation ○ Induced abnormal testicular pathological changes ○ Elevated H_2_O_2_ and MDA levels ○ Reduced testicular SOD activity ○ Decreased spermatogenic cell counts ○ Enhanced caspase-3 activity and increased TUNEL-positive cells, hence promoting apoptosis
[66]	Comparative	Male mice	SO_2_			○ Exposure to SO_2_ induced changes in the social and/or agonistic behavior of these animals ○ They concluded that prenatal SO_2_ exposure can alter mouse social/agonistic behavior
[59]	Experimental	Rats	SO_2_	10 ppm, 4 h/day	16	○ Exposure to SO_2_ resulted in the following: ○ Reduced sperm motility ○ Increased testis weight-to-body weight ○ Abnormal and loose histological arrangement of the spermatogenic cells ○ Increased local structural damage in the seminiferous tubules
[60]	Experimental	Rats	PM_10_, NO_2_, NOx	6 h/day for 5 days/week for 3 months		○ Exposure to the different pollutants caused a reduction in sperm production, and the activity of testicular hyaluronidase was significantly reduced ○ There was a decrease in the number of spermatids count in the seminiferous tubules in stages VI, VII, and VIII tubules in the testes ○ Altered serum sex hormone level was also reported
[61]	Experimental	Rats, Primary spermatogonia, and Leydig cells	PM_2.5_	12 weeks, 24 h	24	○ Animals exposed to PM_2.5_ presented with reductions in the following: ○ Total sperm count ○ Total motility ○ Sperm concentration ○ Sperm viability ○ Primary testicular spermatogonia and Leydig cells cultured with PM_2.5_ (0–320 μg/mL) for 24 h decreased cell survival rate, increased reactive oxygen species, lactate dehydrogenase, and 8-hydroxydeoxyguanosine levels, induced DNA damage, endoplasmic reticulum stress, and apoptosis, and inhibited the secretion and synthesis of testosterone in Leydig cells
[67]	Experimental	Mice, TM4 cells	PM_2.5_			○ Maternal exposure to PM_2.5_ resulted in a decreased number of adult offspring and reduced sperm motility ○ Also observed was increased vacuolization in the Sertoli cells of mice exposed to PM_2.5_ in utero ○ The levels of inhibin and testosterone were reduced and the levels of LH and FSH increased in the PM_2.5_ groups ○ In vitro exposure of TM4 cells to PM_2.5_ resulted in the inhibition of cell cycle and also increased apoptosis
[62]	Experimental	Rats	PM_2.5_	Exposed once every 3 days for 2 months		○ Exposure of rats to varying concentrations of PM_2.5_ during two seasons (winter and summer) resulted in reduced sperm motility and increased percentage of spermatozoa with abnormal morphology ○ Animals in the winter PM_2.5_ group showed severe testicular tissue injury, and the reproductive toxicity of winter PM_2.5_ in the testis was stronger than that of summer PM_2.5_ ○ Animals exposed to PM_2.5_ during the summer and winter showed testicular germ cell apoptosis

## Data Availability

Not applicable.
